# Age-Characteristic Changes of Glucose Metabolism, Pancreatic Morphology and Function in Male Offspring Rats Induced by Prenatal Ethanol Exposure

**DOI:** 10.3389/fendo.2019.00034

**Published:** 2019-02-04

**Authors:** Di Xiao, Hao Kou, Shuxia Gui, Zhenyu Ji, Yu Guo, Yin Wu, Hui Wang

**Affiliations:** ^1^Department of Pharmacology, School of Basic Medical Sciences of Wuhan University, Wuhan, China; ^2^Department of Pharmacy, Zhongnan Hospital, Wuhan University, Wuhan, China; ^3^Hubei Provincial Key Laboratory of Developmentally Originated Diseases, Wuhan, China

**Keywords:** prenatal ethanol exposure, intrauterine growth restriction, pancreatic β cell development, insulin expression, glucocorticoids-insulin-like growth factor 1 axis

## Abstract

Intrauterine growth restricted offspring suffer from abnormal glucose homeostasis and β cell dysfunction. In this study, we observed the dynamic changes of glucose metabolic phenotype, pancreatic morphology, and insulin synthesis in prenatal ethanol exposure (PEE) male offspring rats, and to explore the potential intrauterine programming mechanism of the glucocorticoid-insulin-like growth factor 1 (GC-IGF1) axis. Ethanol (4 g/kg·d) was administered through oral gavage during gestational day (GD) 9–20. Serum glucose and insulin levels, pancreatic β cell mass, and expression of glucocorticoid receptor (GR), IGF1 and insulin were determined on GD20, postnatal week (PW) 6, PW12 with/without chronic stress (CS), and PW24, respectively. Both intraperitoneal glucose and insulin tolerance tests were conducted at PW12 and PW24. Results showed that the serum glucose and insulin levels as well as pancreatic β cell mass were reduced on GD20 in PEE males compared with the controls, while pancreatic GR expression was enhanced but IGF1 and INS1/2 expression were suppressed. After birth, compared with the controls, β cell mass in the PEE males was initially decreased at PW6 and gradually recovered from PW12 to PW24, which was accompanied by increased serum glucose/insulin levels and insulin resistance index (IRI) at PW6 and decreased serum glucose contents at PW12, as well as unchanged serum glucose/insulin concentrations at PW24. In addition, both improved glucose tolerance and impaired insulin sensitivity of the PEE males at PW12 were inversed at PW24. Moreover, at PW6 and PW12, pancreatic GR expression in the PEE group was decreased, while IGF1 expression was reversely increased, resulting in a compensatory increase of insulin expression. Moreover, CS induced pancreatic GR activation and inhibited IGF1 expression, resulting in impaired insulin biosynthesis. Conclusively, the above changes were associated with age and the intrauterine programming alteration of GC-IGF1 axis may be involved in prenatal and postnatal pancreatic dysplasia and impaired insulin biosynthesis in PEE male offspring.

## Introduction

Alcohol consumption is common in both developing and developed countries. Epidemiological studies have shown that the alcoholism rate in young women has increased in recent years (1), and some of female alcoholics could not quit drinking during pregnancy (2). Prenatal alcohol exposure can lead to a range of adverse developmental outcomes in offspring, which are collectively termed fetal alcohol spectrum disorder (FASD) (3). In European countries, the morbidity of FASD is 0.97 per 1,000 births, whereas the morbidity of FASD in U.S. is 1.95 per 1,000 births (4). As one of the primary symptoms of FASD, intrauterine growth restriction (IUGR) is usually diagnosed with a birth weight and/or length below the 10th percentile for gestational age and an abdominal circumference that is less than the 2.5th percentile, with pathologic restriction of fetal growth (5). Human surveys and animal studies indicate that IUGR offspring induced by prenatal ethanol exposure (PEE) are characterized by altered glucose homeostasis and an increased risk of type 2 diabetes (T2DM) in adulthood (6, 7). It has been suggested that PEE induces glucose intolerance and hyperinsulinemia in IUGR male rats on postnatal day (PD) 91 (6, 8) and that the abnormality of glucose metabolism is more substantial in PEE male offspring (9, 10).

Intrauterine environmental challenges can “program” pancreatic β-cell structure and function (11, 12), which lead to glucose-insulin metabolic dysfunction and increased T2DM risk in adult offspring (13). The postnatal dysfunction of metabolic phenotype and abnormal pancreatic morphology induced by PEE have been reported in some studies (6–8, 14). However, these studies have some contradictory results, which might be attributed to a single selected time point for the investigations (15). Therefore, the dynamic changes of pancreatic development and glucose metabolism in PEE offspring, as well as the intrauterine programming mechanism, remains to be clarified.

It has been documented that glucocorticoids (GC) could affect pancreatic differentiation *in utero* (16, 17). High levels of glucocorticoids might induce an imbalance of pancreatic differentiation, reduction of β cell mass, and deceleration of insulin expression and secretion (16, 18). Insulin-like growth factor 1 (IGF1) is one of the key factors regulating pancreatic development (19). IGF1 not only promotes rapid division and proliferation of pancreatic β cells (20, 21) but also suppresses their apoptosis (20, 22). Furthermore, the IGF1 signaling pathway could regulate the proliferation of pancreatic precursor cells to affect the directional differentiation of β cells (23). It has been documented that glucocorticoids could reduce IGF1 expression in various cells via glucocorticoid receptor (GR) activation (24, 25). The evidence mentioned above suggests that the effect of glucocorticoids on IGF1 expression may play an important role in pancreatic development and β cell mass.

Previously, we have demonstrated that ethanol administered though oral gavage on gestational day (GD) 9–20 caused IUGR in rats (26). Furthermore, PEE can induce fetal rat over-exposure to maternal GC, which program IUGR offspring to be susceptible to multiple adult diseases (26–28). It is still unknown whether high levels of glucocorticoids in fetal blood can change the expression of IGF1 in the pancreas and further affect pre- and postnatal pancreatic development and insulin synthesis. In previous studies, we found a negative relationship between serum corticosterone (CORT) levels and IGF1 contents in PEE offspring before and after birth; this negative relationship also correlates to the increased susceptibility to metabolic diseases. Based on these findings, we proposed a programming alteration mechanism of the glucocorticoid-insulin-like growth factor 1 (GC-IGF1) axis to explain multi-organ development toxicity and disease susceptibility in PEE offspring (26, 28).

Researches reveal that IUGR is associated with glucose intolerance in both men and women during adulthood (29), while the female offspring of undernourished rats do not develop glucose intolerance (30) or develop insulin resistance only in old age (31), suggesting gender-specific programming of glucose metabolism in these animals. Evidence also reveals that males are more sensitive to early life programming of insulin action than females (32). In this study, we observed dynamic changes in the glucose metabolic phenotype, pancreatic morphology and insulin synthesis in the PEE male offspring. The selected time points include GD20, postnatal week (PW) 6, 12, and 24, which are approximately equivalent to the fetus, childhood, early, and late stages of adulthood in humans (33). Moreover, a previously described chronic stress (CS) test, induced by a 2-week ice-water swimming test from PW10 (33, 34), was used to confirm the involvement of the GC-IGF1 axis in the alteration of pancreatic development and insulin synthesis induced by PEE. This study is significant for clarifying the developmental toxicity of ethanol and seeking early prevention and treatment strategies for fetal-originated adult diabetes.

## Materials and Methods

### Chemicals and Reagents

Ethanol (analytical pure grade) was purchased from Zhen Xin Co., Ltd. (Shanghai, China). Isoflurane was obtained from Sinopharm Chemical Reagent Co., Ltd. (Shanghai, China). Ultrasensitive Rat insulin ELISA kits, Rat insulin ELISA kits and Rat/Mouse Proinsulin ELISA kits were provided by Mercodia (Uppsala, Sweden). Glucose oxidase assay kits were purchased from Mind Bioengineering Co. Ltd. (Shanghai, China). TRIzol reagent was purchased from Invitrogen Co. (Carlsbad, CA, USA). Reverse transcription and real-time quantitative polymerase chain reaction (RT-qPCR) kits were offered by TaKaRa Biotechnology Co., Ltd. (Dalian, China). Oligonucleotide primers for rat RT-qPCR genes (PAGE purification) were custom synthesized by Sangon Biotech Co., Ltd. (Shanghai, China). RNAlater was provided by Qiagen (Düsseldorf, North Rhine-Westphalia, Germany). Glucocorticoid receptor (GR), IGF1, and insulin gene enhancer protein isl1 (ISL1) antibodies were obtained from Abcam (San Francisco, California, USA). Insulin antibody was provided by Sigma-Aldrich (St. Louis, Missouri, USA). Immunohistochemical staining agents were purchased from Zhongshan Golden Bridge Biotechnology Co. Ltd. (Beijing, China).

### Animals and Treatment

This project was performed at the Center for Animal Experiment of Wuhan University (Wuhan, China), which is recognized and designated by the Association for Assessment and Accreditation of International Laboratory Animal Care. This study was carried out in accordance with the Guidelines for the Care and Use of Laboratory Animals of the Chinese Animal Welfare Committee (AAALAC International). The protocol was approved by the Committee on the Ethics of Animal Experiments of the Wuhan University School of Medicine (permit number: 14016). Specific pathogen free Wistar rats (12~13-week-old, with weights of 197–221 g for females and 241–275 g for males) were purchased from the Experimental Center of Hubei Medical Scientific Academy (No. 2012–2014, certification number: 42000600002258, license number: SCXK [Hubei]).

**Figure 1:**
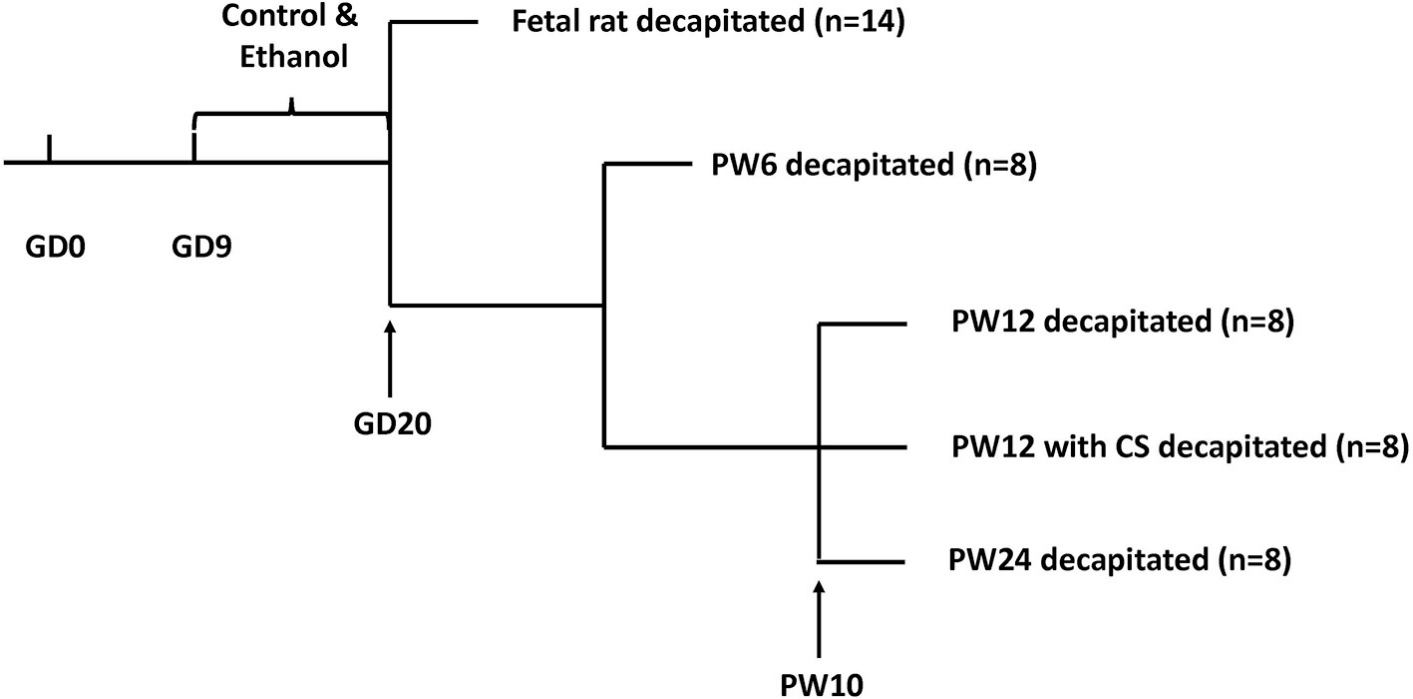
Animal processing schedule. Intrauterine growth restriction modeling was performed using prenatal ethanol exposure (4 g/kg.d) from gestational day (GD) 9 to GD20. Beginning at postnatal week (PW) 10, some pups were subjected to a 5 min ice-water swim (5–7°C) to induce chronic stress. Pups were decapitated at GD20, PW6, PW12, PW12 with chronic stress, and PW24.

Animals were fed in metal wire cages in temperature-controlled conditions and allowed *ad libitum* access to tap water and standard chow at all times (room temperature: 18–22°C; humidity: 40–60%, light cycle: 12 h light-dark cycle; 10–15 air changes per hour). After 7 days of acclimation, each two female rats and one male rat were mated from 7:00 p.m. to 7:00 a.m. The appearance of a vaginal plug or vaginal smear with sperm cells was confirmed as successful mating. We designated that day as GD0. Pregnant rats were then transferred to individual cages. From GD9 to GD20, the pregnant rats were given ethanol (4 g/kg·d, 40%) or distilled water through oral gavage at 8:00 a.m. every day as described in previous studies (26, 35). The animal processing schedule was shown in Figure 1.

For fetal rat experiment, a portion of pregnant rats were anesthetized with isoflurane and decapitated, and fetal rats were obtained through cesarean section on GD20. To eliminate the interferences induced by litter size, only pregnant rats with 8~14 pups were included and each group finally contained 14 pregnant rats. The fetal rats were quickly picked and weighed. Fetal rats were decapitated, and the pancreases were weighed. The fetal blood or pancreases (per litter) were collected and pooled into one independent sample, respectively. Fetal blood was centrifuged at 2263 × g for 15 min, and the serum was stored at −80°C. Some intact fetal pancreases (including the head, body and tail) were weighed and put into RNAlater (1 mg: 100 μl) overnight at 4°C, and then transferred to the −80°C refrigerator for gene expression analysis. Other intact fetal pancreases (head, body, and tail) were selected and fixed per litter (*n* = 5) for histology observation or fixed for electron microscope observation.

For postnatal rat experiment, the remaining pregnant rats (including the control and PEE groups) were subjected to delivery. The pups remained with their original mothers after birth and throughout lactation. To ensure adequate and relatively equal intake of nutrients during the suckling period, only litters with 8~14 pups were chosen for the following experiment and normalized to 8 pups per litter (male: female = 1:1) (36). Finally, a total of 16 litters (8 controls & 8 PEEs) were included and all pups were weaned until postnatal week (PW) 4. Four male offspring rats from each litter were selected and assigned to the four studies at different postnatal time points or condition (i.e., PW6, PW12, PW12 with CS and PW24), respectively. The CS test was achieved by forced 5-min ice-water swimming per day (5–7°C) from PW10 to PW12 (33, 34). Intraperitoneal glucose tolerance tests (IPGTT) and insulin tolerance tests (ITT) were performed at PW12 and PW24. Pups were anesthetized with isoflurane and then decapitated. Blood and pancreases were collected. Intact pancreases (including head, body, and tail) were weighed. Blood was centrifuged at 2263 × g for 15 min, and the serum was stored in an −80°C refrigerator for gene expression analysis. Some pancreases were weighed and put into RNAlater (1 mg: 100 μl) overnight at 4°C, and then they were restored at −80°C. Other pancreases (*n* = 5) were fixed in a 10% neutral formalin solution for histology observation.

### IPGTT, ITT, and Area Under the Curve (AUC)

Based on the reference (37), rats were pre-fasted for 12 h before the IPGTTs, which started at 8:00 a.m. Each rat was intraperitoneally administered glucose (2 g/kg). The interval time of administration to each animal was 2 min, and the total operating time was limited to within 30 min. Blood glucose levels which measured 0, 15, 30, 60, and 120 min after the glucose challenge were determined using a glucometer (ACCU-CHEK Performa, Roche). The AUCs were calculated using the trapezoidal rule (38). ITTs were administered after a 5 h fast (8:00 a.m.−1:00 p.m.) (39), and insulin (1 unit/kg) was intraperitoneally injected. The measurement of blood glucose levels which measured 0, 15, 30, 60, and 120 min after the insulin challenge and calculation of the corresponding AUCs was the same as that described for the IPGTTs. Con. N min represented serum glucose at each time point. Time N min-N min represented the time gap between two time points.

Con. N_min_ (%) = Con. N_min_ / Con. 0_min_AUC = (Con. 0_min_+Con. 15_min_)/2 × Time 0_min_-15_min_+ (Con. 15_min_+Con. 30_min_)/2 × Time 15_min_-3_min_+(Con. 30_min_+Con. 60_min_)/2 × Time 30_min_-60_min_+(Con. 60_min_+Con. 120_min_)/2 × Time 60_min_-120_min_

### Serum Glucose/Insulin Concentrations and Pancreatic Insulin/Proinsulin Contents Determination

The serum insulin and glucose levels were measured through ELISA and biochemical assay kits, respectively, following the manufacturer's protocol. The fasting glucose (FG), fasting insulin (FI) and insulin resistance index (IRI) was acronymized as b. Pancreatic insulin and proinsulin were extracted with acidic ethanol (1.5% [vol/vol] HCl in 75% [vol/vol] ethanol) and assayed using ELISA kits.

IRI = FG (mmol/L) × FI (mIU/L)/22.5

### Pancreatic Morphometric Analysis

Pancreases at GD20, PW6, PW12, and PW24 were weighed and fixed in 10% neutral buffered formalin overnight, dehydrated, and embedded in paraffin. Each analytical result of the pathological indices was obtained from a total of five animals per group, with five complete longitudinal sections (5 μm) per embedded pancreas (i.e., at their maximum widths) in (at minimum) 200 μm intervals. The hematoxylin-eosin (HE) and insulin immunohistochemical staining were subjected to the pancreases of GD20 to PW24. As a negative control, pancreatic sections underwent similar treatment, and no positive reactions to the antibody against insulin were observed (data not shown). Sections were viewed at a magnification of ×40. Approximately 3–4 fields per fetal pancreas section and 30–40 fields per postnatal pancreas section were acquired, and we used NIS-Elements Br 4.20 (Nikon, USA) to analyze the total pancreas areas and β-cell areas (insulin-positive cells). The size of the insulin-positive cell area and the pancreatic tissue area were measured (average value, five sections per animal). The β cell fraction and mass were calculated according to the formula provided below (40). The unit of β cell mass/body weight was μg/g (39). Fetal pancreases were cut and fixed with 2.5% glutaraldehyde in 0.1 M phosphate buffer (pH 7.4) for 2 h at 4 °C and post fixed with 1% osmium tetroxide. We dehydrated these tissues with a graded series of ethanol and then embedded them in Epon 812. Ultrathin sections (~50 nm) were cut with LKB-V ultramicrotome (Bromma, Sweden), dual stained with uranyl acetate and lead citrate and observed with a Hitachi H600 transmission electron microscope (EM) (Hitachi, Tokyo, Japan).

β cell fraction (%) = β cell area/total pancreas area × 100

β cell mass (mg) = β cell fraction × pancreas weight.

### Pancreatic mRNA and Protein Expression Analysis

GD20 and PW6 pancreases (including the head, body, and tail) were grinded and mixed in liquid nitrogen. We used 25 mg of pancreatic tissue to extract total RNA, based on the manufacturer's protocol (RNA-Solv Reagent). The concentrations and purity of total RNA were measured in *A*_260*nm*_ and *A*_280*nm*_, and the rates of *A*_260*nm*_ */A*_280*nm*_ were kept between 1.8 and 2.0. The total RNA concentrations were adjusted to 1 μg/μL. cDNA synthesis and RT-qPCR amplification were performed using the manufacturer's protocol. Relative standard curves were constructed for the following target genes: GR, IGF1, ISL1, insulin 1 (INS1), and INS2. The oligonucleotide primers and annealing temperatures in RT-qPCR are listed in Table 1. To precisely quantify the gene transcripts, the mRNA level of the housekeeping gene glyceraldehyde phosphate dehydrogenase (GAPDH) was measured and used as the quantitative control (26, 35).

**Table 1:** Oligonucleotide primers and PCR conditions of rat in quantitative real-time PCR.

**Genes**	**Forward primer**	**Reverse primer**	**Accession number**	**Product (bp)**	**Annealing (°C)**
ISL1	ACTGAGTGACTTCGCCTTGC	ATCTGGGAGCTGAGAGGACA	NM_017339.3	133	60
INS1	TCAGCAAGCAGGTCATTGTT	AGGTACAGAGCCTCCACCAG	NM_019129.3	147	57
INS2	TCTTCTACACACCCATGTCCC	GGTGCAGCACTGATCCAC	NM_019130.2	149	55
IGF1	GACCAAGGGGCTTTTACTTCAAC	TTTGTAGGCTTCAGCGGAGCAC	NM_178866.4	148	60
GR	CACCCATGACCCTGTCAGTG	AAAGCCTCCCTCTGCTAACC	NM_012576.2	156	54
GAPDH	GCAAGTTCAACGGCACAG	GCCAGTAGACTCCACGACA	NM_017008.4	140	60

*ISL1, insulin gene enhancer protein isl1; INS1, insulin 1; INS2, insulin2; IGF1, insulin-like growth factor 1; GR, glucocorticoid receptor; GAPDH, glyceraldehyde-3-phosphate dehydrogenase*.

The relative protein expressions of GR, IGF1, ISL1, and insulin in PW12 and PW24 were semi-quantified by immunohistochemistry (IHC) and the area of these protein expression in the pancreatic tissues also could be observed. PW12 and PW24 pancreases were embedded and sliced as mentioned above. As negative controls, pancreatic sections underwent similar treatment, and no positive reactions to the antibody against GR, IGF1, ISL1, and insulin were observed (data not shown). The mean density for pancreases in the same group was taken as the expression level for the protein of interest. Sections were viewed at a magnification of ×400. Approximately 200 fields per postnatal pancreas section were acquired, and we used NIS-Elements Br 4.20 (Nikon, USA) to analyze the mean density of GR, IGF1, ISL1, and insulin staining.

### Statistical Analysis

All data presented were expressed as the mean ± S.E.M., and the statistical analysis was performed with SPSS for Windows version 19 (SPSS Science Inc., Chicago, IL, USA). The normality and homogeneity of the variances were analyzed using the one-sample Kolmogorov–Smirnov test and Levene's test, respectively. Differences between the control and PEE groups were compared using a repeated measures ANOVA (IPGTT and ITT) or two-tail Student's test. Groups which were assumed to have normal distribution, and equal variances were compared through repeated measures ANOVA and Student's two-tailed *t*-test. When data failed tests for normal distribution and homogeneity of variance, we chose the Mann–Whitney U test (equivalent to Wilcoxon W). Statistical significance was designated at *P* < 0.05 and *P* < 0.01.

## Results

### In Male Fetal Rats

#### Islet Function and Pancreatic Morphological Changes

**Figure 2:**
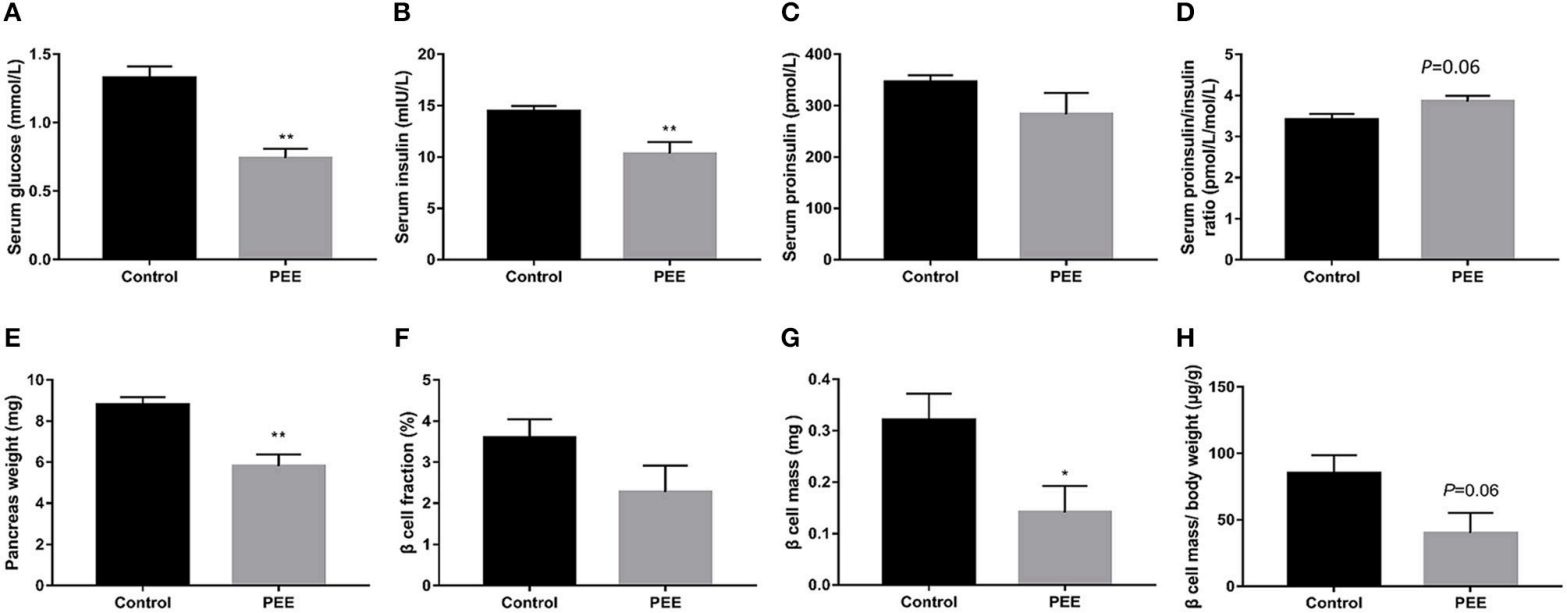
Effects of prenatal ethanol exposure (PEE) on islet function and pancreatic morphology in male fetal rats. **(A)** Serum glucose, **(B)** serum insulin, **(C)** serum proinsulin, **(D)** ratios of serum proinsulin/insulin, **(E)** pancreas weight, **(F)** pancreatic β cell fraction, **(G)** pancreatic β cell mass, **(H)** pancreatic β cell mass/ body weight ratio, Mean ± S.E.M., *n* = 14 for fasting glucose serum and insulin. *P < 0.05, **P < 0.01 vs. control. The representative images of pancreatic insulin immunohistochemical staining (×40 and ×400) were shown in Supplementary Figures 1A–D.

In our previous study, a decrease bodyweight and a high IUGR rate were observed in PEE male fetal rats (26). Based on this batch of animals, we further observed changes in islet function and pancreatic morphology of fetal rats. Compared with the controls, serum glucose and insulin levels of the PEE fetal rats were decreased (*P* < 0.01, Figures 2A,B), while serum proinsulin level was unchanged (Figure 2C), as a result, the ratio of serum proinsulin and insulin presented increasing trend in the PEE offspring (*P* = 0.06, Figure 2D). Pancreas weight (*P* < 0.01, Figure 2E), β cell mass (*P* < 0.05, Figure 2G), and β cells mass/body weight (*P* = 0.06, Figure 2H) were all decreased or showed a decreasing trend in the PEE fetal rats, whereas the β cell fraction (Figure 2F) did not change. The typical β cell ultrastructure could be observed in the fetal pancreases from the control group (Supplementary Figures 1E,F): the mature dense-core β-granules were highly populated, and there were halo spaces between the core and β-granule membrane. However, there was lower electron density and a lack of the characteristic halos of mature β-granules in the PEE group. The above data suggest that islet function was reduced, and pancreatic morphology was impaired in the PEE male fetal rats.

#### Pancreatic Insulin Biosynthesis, GR and IGF1 Expression Changes

**Figure 3:**
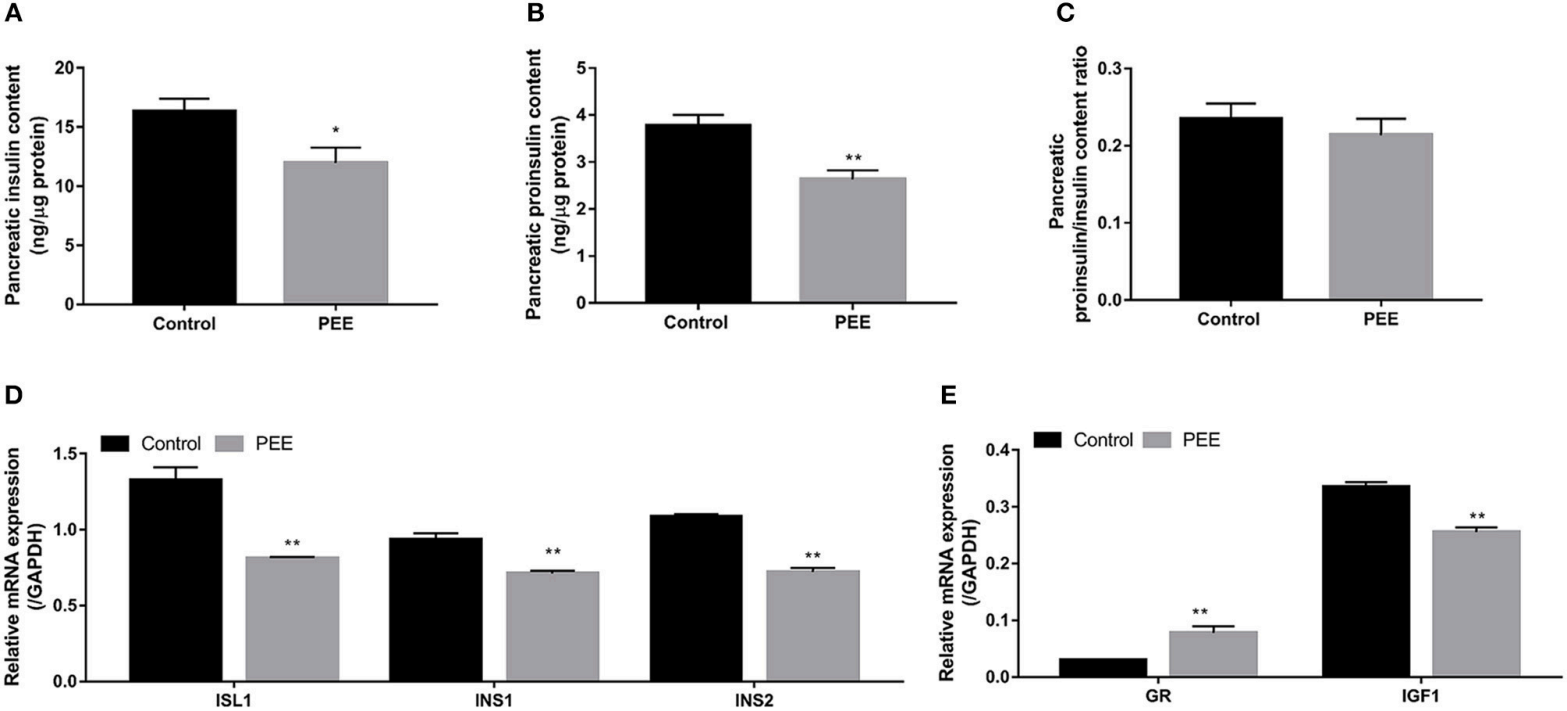
Effects of prenatal ethanol exposure (PEE) on pancreatic insulin synthesis, mRNA expression of glucocorticoid receptor (GR), and insulin-like growth factor 1 (IGF1) in male fetal rats. **(A)** Insulin contents, **(B)** proinsulin contents, **(C)** ratios of proinsulin/insulin, **(D)** mRNA expression of insulin gene enhancer protein isl1 (ISL1), insulin 1 (INS1) and INS2, and **(E)** gene expression of GR and IGF1. Mean ± S.E.M., *n* = 14. *P < 0.05, **P < 0.01 vs. control.

The results indicated that the contents of pancreatic insulin and proinsulin in the PEE group were lower than those in the controls (*P* < 0.05, *P* < 0.01, Figures 3A,B), whereas the ratio of proinsulin/insulin was unchanged (Figure 3C). Furthermore, compared with the controls, the expression levels of ISL1, INS1, and INS2 mRNA were decreased in the PEE fetal pancreases (*P* < 0.01, Figure 3D). Meanwhile, the GR mRNA expression level was increased, while the expression level of the IGF1 gene was decreased (*P* < 0.01, Figure 3E). These results suggest that pancreatic insulin biosynthesis was inhibited and that this inhibition was accompanied by increased Gr expression and decreased IGF1 expression.

### In Male Offspring Rats at Different Time Points After Birth

#### Glucose Metabolic Phenotype Changes at Different Time Points

**Figure 4:**
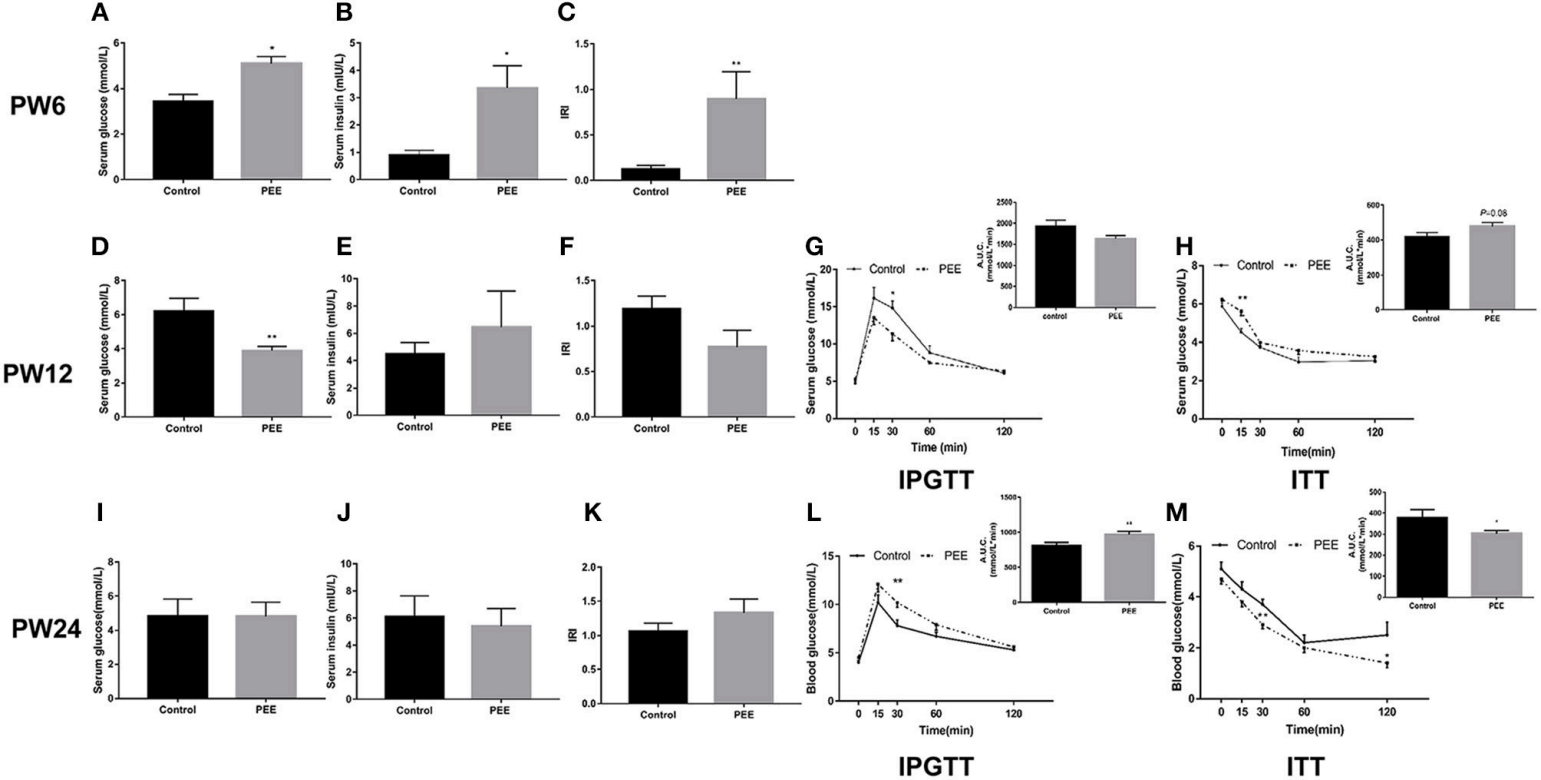
Effects of prenatal ethanol exposure (PEE) on the glucose metabolism phenotype at postnatal week (PW) 6, PW12, and PW24 male offspring rats. **(A–C)** Serum glucose and insulin levels, as well as insulin resistance index (IRI) at PW6, **(D–F)** serum glucose and insulin levels, as well as IRI at PW12, **(G)** blood glucose concentrations during intraperitoneal glucose tolerance test (IPGTT) and area under the curve (AUC) at PW12, **(H)** blood glucose concentrations during insulin tolerance test (ITT) and the AUC at PW12, **(I–K)** serum glucose and insulin levels as well as IRI at PW24, **(L)** blood glucose concentrations during IPGTT and AUC at PW12, and **(M)** blood glucose concentrations during ITT and AUC at PW24. Mean ± S.E.M., *n* = 8. *P < 0.05, **P < 0.01 vs. control.

At PW6, serum glucose, insulin levels and IRI were increased in the PEE group compared with the controls (*P* < 0.05, *P* < 0.01, Figures 4A–C). However, at PW12, the basal level of serum glucose was significantly decreased (*P* < 0.01, Figure 4D), while the serum insulin concentration and IRI did not change in the PEE group (Figures 4E,F). In the IPGTT of the PEE group, when compared with the controls, the serum glucose concentration at 30 min was lower in the PEE male pups (*P* < 0.05, Figure 4G), while the corresponding AUC was not obviously changed. In the ITT of the PEE group, the 15 min serum glucose concentration was higher than that of the control (*P* < 0.01), whereas the corresponding AUC in the PEE group was 1.13-fold higher than that of the controls (*P* = 0.08, Figure 4H). These results suggest that the glucose tolerance was increased, while the insulin sensitivity was decreased in the PEE male pups of PW12. At PW24, the basal serum glucose and insulin levels, as well as the IRI of the PEE male rats, were not altered (Figures 4I–K). However, the glucose tolerance of the PEE offspring was remarkably weakened compared with the control, as evidenced by the conspicuous increase in the concentration of blood glucose at 30 min and the increased corresponding AUC after adding the glycemic load (*P* < 0.01, Figure 4L). Furthermore, in the ITT, the serum glucose levels of the PEE males at 30 and 120 min, as well as the corresponding AUC, were decreased (*P* < 0.01, *P* < 0.05, Figure 4M). These data suggest that a weakened glucose intolerance and enhanced insulin sensitivity existed, although there was no significant change in the glucose metabolic phenotype in the PEE male offspring rats for PW24.

#### Pancreatic Morphological Changes at Different Time Points

**Figure 5:**
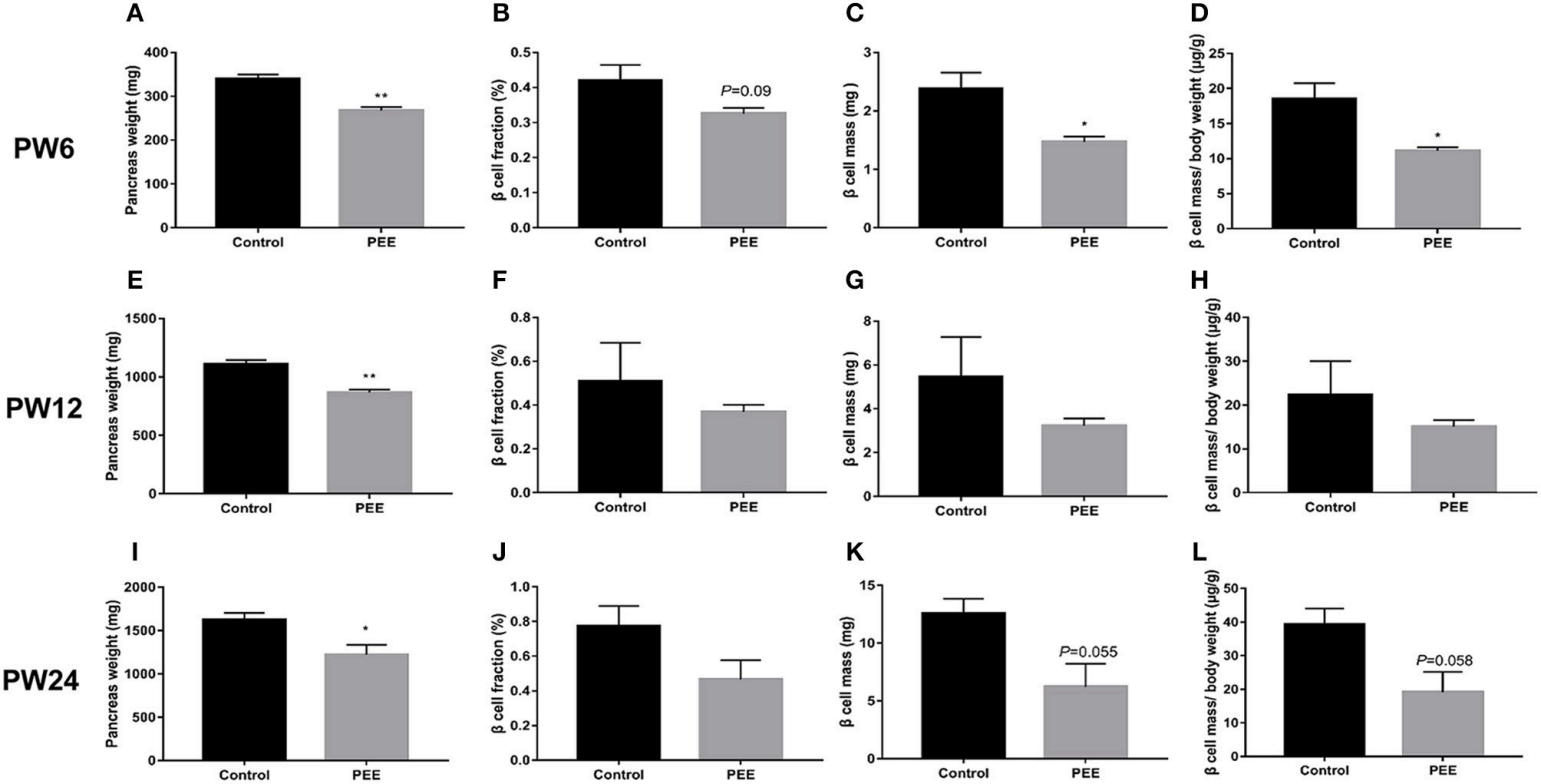
Effects of prenatal ethanol exposure (PEE) on the pancreatic morphology of offspring male rats at postnatal week (PW) 6, PW12 and PW24. **(A)** pancreas weight at PW6, **(B)** β cell fraction at PW6, **(C)** β cell mass at PW6, **(D)** β cell mass/body weight rates at PW6, **(E)** pancreas weight at PW12, **(F)** β cell fraction at PW12, **(G)** β cell mass at PW12, **(H)** β cell mass/body weight rates at PW12, **(I)** pancreas weight at PW24; **(J)** β cell fraction at PW 24, **(K)** β cell mass at PW 24, and **(L)** β cell mass/body weight rates at PW24. Mean ± S.E.M., *n* = 5. *P < 0.05 **P < 0.01 vs. control. The representative images of pancreatic insulin immunohistochemical staining (×40) were shown in Supplementary Figures 2A,B,E,F,I,J.

At PW6, compared with the controls, the pancreas weight (*P* < 0.01, Figure 5A), β cell fraction (*P* = 0.088, Figure 5B), β cell mass (*P* < 0.05, Figure 5C), and β cell mass/body weight rate (*P* < 0.05, Figure 5D) were all decreased or showed a decreasing trend in the PEE offspring. However, at PW12, the β cell fraction (Figure 5F), β cell mass and β cell mass/body weight rate (Figures 5G,H) showed no obvious changes, except for the lower pancreas weight (*P* < 0.01, Figure 5E). At PW24, the pancreas weight (*P* < 0.05, Figure 5I) was still decreased, while the β cell mass (*P* = 0.055, Figure 5K) and β cell mass/body weight rate (*P* = 0.058, Figure 5L) only showed a decreasing tendency in the PEE group, and the β cell fraction did not show any obvious changes (Figure 5J). The above data suggest that β cells demonstrate a “catch-up” growth pattern in the PEE male offspring.

#### Pancreatic Insulin Biosynthesis, GR and IGF1 Expression Changes at Different Time Points

**Figure 6:**
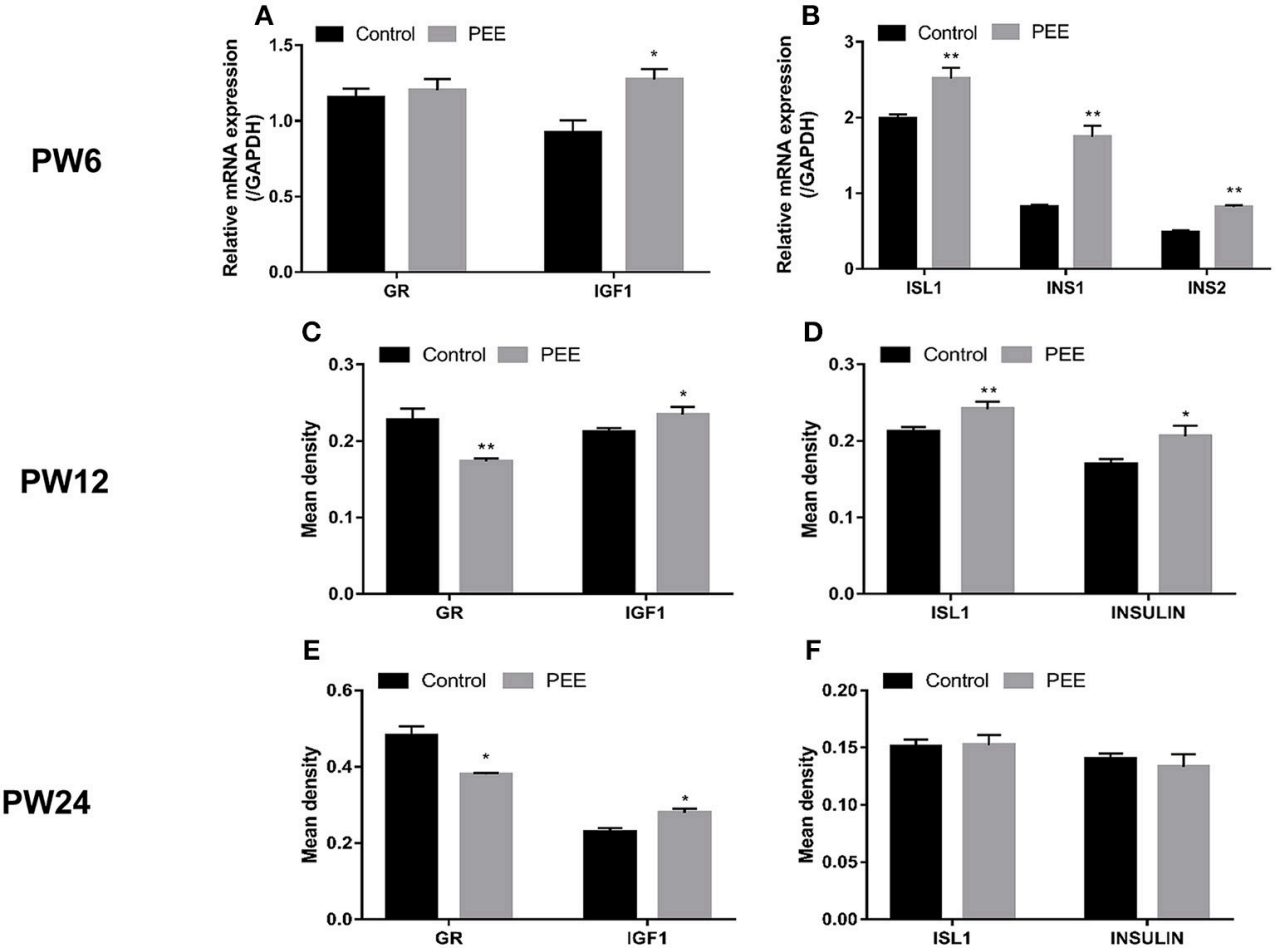
Effects of prenatal ethanol exposure (PEE) on the expression of pancreatic insulin, glucocorticoid receptor (GR) and insulin-like growth factor 1 (IGF1) at postnatal week (PW) 6, PW12, and PW24 male offspring rats. **(A,B)** mRNA expression levels of GR, IGF1, insulin gene enhancer protein isl1 (ISL1), insulin1 (INS1) and INS2 at PW6, **(C,D)** the mean density of the immunohistochemistry of GR, IGF1, ISL1 and insulin at PW12, **(E,F)** the mean density of immunohistochemistry of GR, IGF1, ISL1 and insulin at PW24. Mean ± S.E.M., *n* = 5. *P < 0.05, **P < 0.01 vs. control. The representative images of pancreatic GR, IGF1, and ISL1 immunohistochemical staining (×400) were shown in Supplementary Figure 3 and the representative images of pancreatic INSULIN immunohistochemical staining (×400) were shown in Supplementary Figures 2C,D,G,H,K,L.

At PW6, when compared with the respective controls, the GR mRNA expression was unchanged while the IGF1 mRNA expression was increased (*P* < 0.05, Figure 6A), meanwhile, the expression levels of ISL1, INS1 and INS2 mRNA were significantly increased (*P* < 0.01, Figure 6B). At PW12 (Figures 6C,D), the GR protein expression level was decreased, however, the IGF1 protein expression level was increased in the PEE offspring rats (*P* < 0.01, *P* < 0.05), and the expression levels of ISL1 and insulin protein were increased (*P* < 0.01, *P* < 0.05). At PW24 (Figures 6E,F), the GR protein expression level was decreased, while that of the IGF1 protein was still increased (*P* < 0.05, *P* < 0.05), however, the protein expression levels of ISL1 and insulin were not significantly changed. These results suggest that the insulin synthesis was enhanced after birth in the PEE male offspring and that this enhancement was accompanied by the down-regulated expression of GR and the up-regulated expression of IGF1 and ISL1. Finally, the expression levels of ISL1 and insulin decreased to the control levels during the late stage of adulthood.

#### Glucose Metabolic Phenotype, Pancreatic GR/IGF1 Expression, and Insulin Biosynthesis Changes at PW12 After a Chronic Stress Test

**Figure 7:**
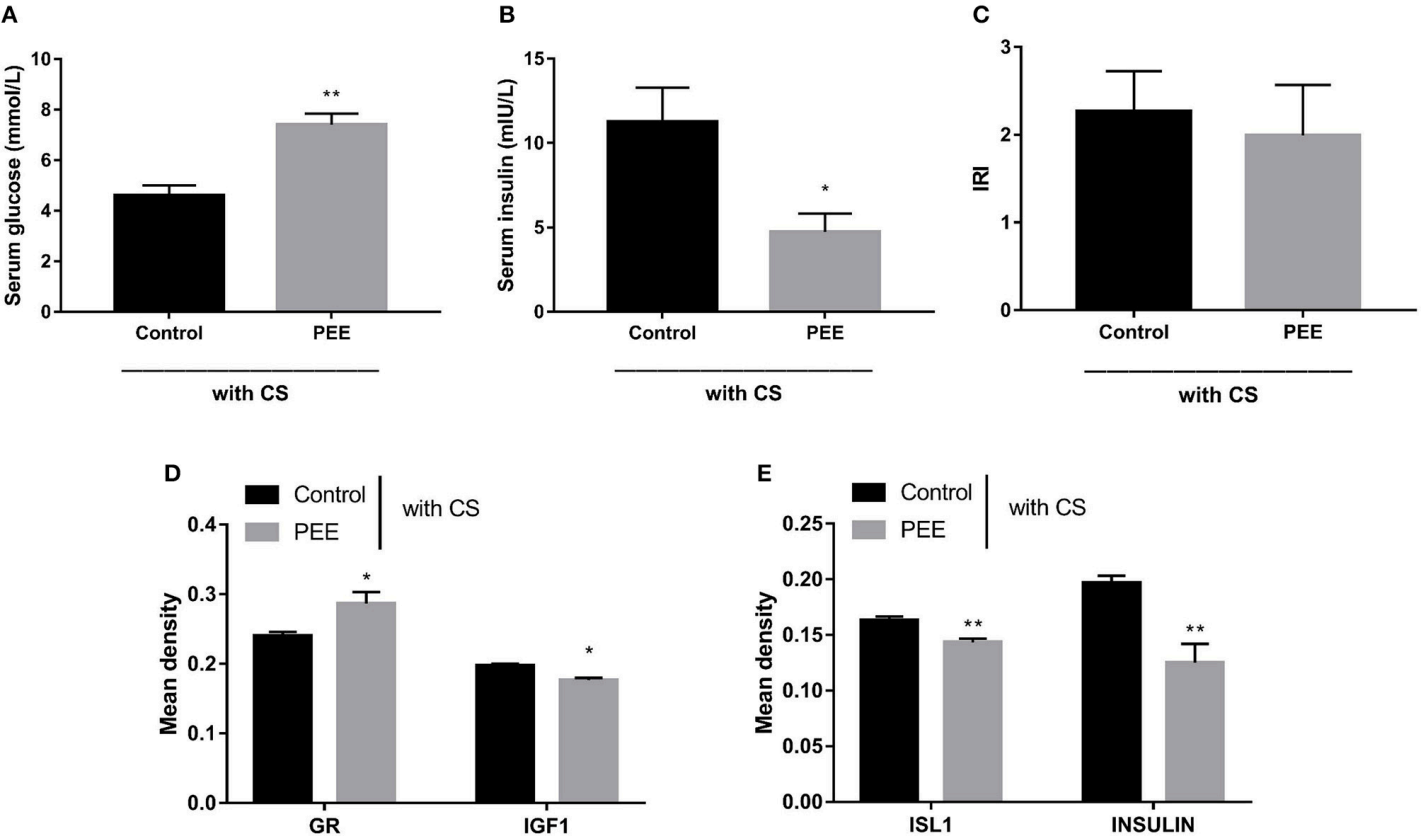
Effects of prenatal ethanol exposure (PEE) on the glucose metabolic phenotype and the expression levels of pancreatic insulin, glucocorticoid receptor (GR), and insulin-like growth factor 1 (IGF1) at postnatal week (PW) 12 in male offspring rats undergoing chronic stress (CS). **(A,B)** Fast serum glucose and insulin, **(C)** insulin resistance index (IRI), **(D,E)** the mean density of immunohistochemistry of glucocorticoid receptor (GR), IGF1, insulin gene enhancer protein isl1 (ISL1) and insulin, Mean ± S.E.M., *n* = 5. *P < 0.05, **P < 0.01 vs. control. The representative images of pancreatic GR, IGF1, ISL1 and INSULIN immunohistochemical staining (×400) were shown in Supplementary Figure 4.

Furthermore, we aimed to confirm the involvement of the GC-IGF1 axis in pancreatic development and insulin synthesis in the PEE offspring. The results showed that, in the PEE group subjected to the CS, the serum glucose level was higher, while the serum insulin level was lower (*P* < 0.01, *P* < 0.05, Figures 7A,B), and the IRI was unchanged (Figure 7C). Meanwhile, the protein expression level of GR was higher, and those of IGF1, ISL1 and insulin were all decreased, compared with the controls (*P* < 0.05, *P* < 0.01, Figures 7D,E).

## Discussions

### Age-Characteristic Changes of Glucose Metabolic Abnormalities in Male PEE Offspring Rats

We have previously demonstrated that PEE causes a reduced birth weight and a high IUGR rate (26). Based on this batch of animals, in the present study, we found that the basal serum glucose and insulin levels of the PEE male offspring rats were decreased at GD20, however, transient insulin resistance appeared at PW6, manifesting as increased basal serum glucose and insulin levels and IRI, which is similar to the finding of age-dependent insulin resistance in a childhood cohort study (41). At PW12, the basal level of serum glucose was remarkably diminished, however, the basal level of serum insulin showed no significant change, and the glucose tolerance was increased, while the insulin sensitivity was decreased in the PEE male offspring. At PW24, the basal levels of serum glucose and insulin were not changed, however, weakened glucose intolerance and enhanced insulin sensitivity were found in the PEE male offspring. The evidences described above confirmed the abnormalities of glucose metabolic phenotype before and after birth, glucose tolerance and insulin sensitivity after birth in the PEE male offspring rats, which were age-characteristic changes and at least partially resulted from pancreatic dysplasia.

### The Abnormalities of Pancreatic β Cell Morphology and Function in Male PEE Fetal Rats

As the sole source organ of insulin, pancreas plays an important role in the regulation of glucose metabolism. Multiple adverse intrauterine environments can induce developmental programming alterations of offspring's pancreas, further affecting its insulin synthesis (40, 42–44). In clinical studies (45–47) and IUGR animal model of food restriction (11, 48), low-protein diet and placenta-uterine deficiency (49–51), a reduction in β cell mass could often be observed. In the present study, the fetal serum glucose and insulin levels were decreased in the male fetal rats of PEE. Meanwhile, the damages in islet morphology and the EM images also suggested that PEE resulted in pancreatic morphological and insulin synthetic function abnormalities in the male fetal rats.

It has been suggested that “fetal over-exposure to maternal glucocorticoids” is one of the important factors contributing to the permanent changes in fetal structure, physiology and metabolism (52). *In vivo* and *in vitro* experiments have shown that dexamethasone can induce pancreatic dysplasia, reduce cell number, and decrease insulin expression and secretion (16). Glucocorticoids could repress IGF1 expression in multiple types of organs and cells via GR activation (24, 25), and decreased IGF1 expression could further inhibit the expression of ISL1 (53), which is one of the most important transcription factors involved in the regulation of pancreatic development and insulin expression (54). In our previous researches, we have demonstrated that PEE could induce fetal over-exposure to maternal glucocorticoids *in utero* (26, 55, 56), which further increased the GR expression [e.g., hippocampus (56) and adrenals (26)] and decreased IGF1 expression [e.g., adrenals (26), and liver (28)] of multiple fetal organs, and thus resulted in their developmental programming alterations. In addition, in other IUGR models induced by prenatal exposure to xenobiotics [such caffeine (57), nicotine (58)], we have also found the fetal over-exposure to maternal glucocorticoids, and the alterations of GR expression in multiple fetal organs, accompanied with related developmental programming changes. In the present study, we found that the contents of pancreatic insulin and proinsulin were lower, and the expression levels of INS1, INS2 and ISL1 mRNA were decreased in the PEE group, furthermore, the GR mRNA expression level was increased but the expression level of IGF1 mRNA was decreased. These results suggested that the lower insulin biosynthesis was likely associated with the pancreatic increased GR expression and decreased IGF1 expression by PEE-induced fetal over-exposure to maternal glucocorticoids.

### The Postnatal Changes of Glucose Homeostasis, Pancreatic β Cell Morphology and Function in Male PEE Offspring Rats

The changes of pancreatic β cell morphology and function induced by adverse intrauterine environments not only appeared *in utero* but also emerged after birth (40, 59, 60). In the present study, the β cell mass of PEE fetal rats were significantly decreased, however, this reduction of β cell population became progressively less obvious after birth. In addition, both serum insulin levels and the insulin gene expression of the PEE offspring were not lower than those of the controls at all selected postnatal time points, which was accompanied by persistent enhanced pancreatic IGF1 expression. As we know, IGF1 is important in regulating β cell growth (61) and usually mediates the postnatal catch-up growth pattern in IUGR offspring (62). Therefore, we presumed that this persistent enhanced pancreatic IGF1 expression might contribute to the postnatal dynamic changes of pancreatic β cell morphology and function in the PEE offspring.

Glucose tolerance relies on several important aspects, including β cell mass and function-associated insulin secretion, as well as insulin availability of the targeted organs (63). At PW12, for the PEE male offspring, although the basal serum insulin level showed no significant changes, the basal serum glucose level was remarkably diminished, while the glucose tolerance was increased but the insulin sensitivity was decreased, suggesting that the sensitivity of peripheral tissues to endogenous insulin might be higher than the sensitivity to exogenous insulin. However, at PW24 in the PEE male offspring, the glucose tolerance was impaired while the insulin sensitivity seems to be improved, suggesting that the sensitivity of peripheral tissues to endogenous insulin might be lower than that to exogenous insulin in the late stage of adulthood. A previous study showed that total insulin and IGF1 resistance in pancreatic β cells causes overt diabetes (64). Therefore, we speculated that the progressive glucose intolerance of PEE offspring from PW12 to 24 might be due to the loss of sensitivity to endogenous insulin and IGF1.

### GC-IGF1 Axis Programming Mechanism Might May be Involved in Age-Characteristic Changes of Pancreatic β Cell Morphology and Function Induced by PEE

Glucocorticoids are a class of key metabolic hormones regulating fetal growth and development as well as the organ maturity *in utero*, while IGF1 plays an insulin-like growth promoting role and is the main factor contributes to IUGR and postnatal catch-up growth (65, 66). In a series of our previous studies, we have demonstrated that PEE induces fetal overexposure to maternal glucocorticoids. The excessive circulatory glucocorticoids in fetus not only inhibits the functional development of HPA axis (67), but also alters the structure and function of multiple fetal multi-organs [i.e., liver (28) and adrenal (26)] via local GR activation and the downstream IGF1 signaling suppression. Furthermore, these changes, particularly the negative regulatory relationship between circulatory glucocorticoids (accompany with GR expression) and IGF1 signaling in various tissues, could be maintained after birth and may contribute to organ dysfunction and lead to enhanced susceptibility to adult diseases. Basing on the above, we proposed the “GC-IGF1 axis programming” mechanism might be involved in the PEE-induced multi-organ dysfunction and predisposition to adult diseases. In the present study, we also observed that the alterations of local pancreatic glucocorticoid action mediated by GR expression presented a negative relationship with changes of pancreatic IGF1, ISL1, and insulin expressions as well as insulin secretion in PEE offspring rats before (GD20) and after birth (PW12 and 24).

Recently, we have demonstrated that PEE programs the hypersensitivity of hypothalamic-pituitary-adrenal (HPA) axis of PW12 male offspring rats, presenting by the increased levels of circulatory CORT after the CS (67). Basing on this, the previously well-described CS model was employed to further verify the negative regulatory relationship between circulatory glucocorticoid (accompany with GR expression) and IGF1 signaling in pancreas and its influence on insulin biosynthesis function in PEE males. Here we found the pancreatic IGF1, ISL1, and insulin expressions of PEE males at PW12 were increased as well as the insulin secretion (Figure 6) which might be attributed to the weaken local glucocorticoid action evidenced by both unchanged CORT levels (67) and decreased pancreatic GR expression. Furthermore, for these PEE male offspring characterized by programmed hypersensitivity of HPA axis, the CS-induced elevation of circulatory CORT activated pancreatic GR, which further inhibits the downstream pancreatic IGF1 and ISL1 expressions thereby causing dysfunction of insulin biosynthesis in PEE males (Figure 7). To sum up, these results observed in PW12 animals without/with CS just collectively revealed the negative regulatory relationship between local glucocorticoid action and IGF1 signaling in pancreas as well as the corresponding influence on insulin biosynthesis. Accordingly, we proposed that “GC-IGF1 axis programming” mechanism maybe mainly mediate the dysfunction of insulin biosynthesis after birth.

It should be noted that, in this study, the degradation of adult pancreatic tissues caused by ineligible preserve condition made RT-qPCR cannot be applied to accurately quantify the relative mRNA expression for GR, IGF1, ISL1 and insulin in pancreatic tissue of PW12 and PW24 animals. Therefore, IHC was chosen to semi-quantify these relative protein expression.

### Ethanol May Also Have Direct Toxic Effects on the Pancreatic Development in Offspring

A series studies reveal that ethanol may also have direct toxic effects on the pancreatic development and function. The research from Kim et al (68) suggested that in PW7 mice, chronic ethanol exposure could induce down-regulation in glucokinases, thus induced β cell apoptosis and declined the β cell function. Researches in β-cell lines (69) and isolated murine islets (70) also suggested that ethanol reduced insulin content and caused ER stress, and resulted in β cell death by apoptosis. In the present study, we found the pancreatic proinsulin/insulin ratios of PEE male fetuses were unchanged with an equally decreased contents of pancreatic insulin and proinsulin, which may be due to the direct toxic effect of EtOH on the β cell function by reducing the production of all proteins including proinsulin and insulin equally. We should further study it in the future work.

## Conclusion

**Figure 8:**
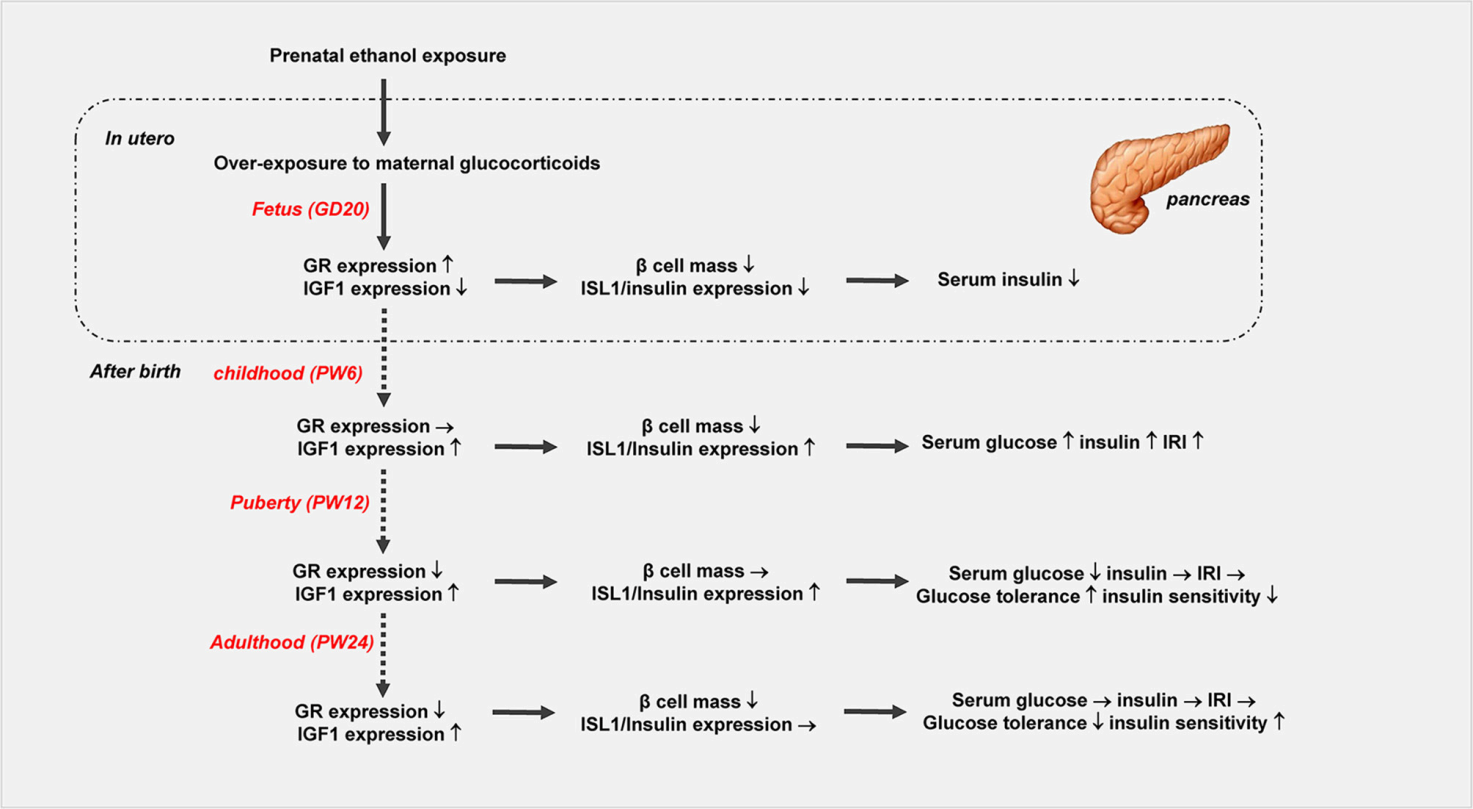
Prenatal ethanol exposure (PEE) induced dynamic changes of pancreatic development and glycometabolism in male offspring rats. GR, glucocorticoid receptor; ISL1, insulin gene enhancer protein isl1; IGF1, insulin-like growth factor 1.

In summary, we systematically confirmed the changes in glucose metabolism and pancreatic dysfunction before and after birth in the PEE male offspring rats with different ages, and we propose here for the first time a “GC-IGF1 axis programming” mechanism maybe mainly mediate the prenatal and postnatal pancreatic dysplasia and dysfunction of insulin biosynthesis (Figure 8). This study will help to clarify the pathophysiological mechanism and explore early prevention and treatment strategies for fetal-originated adult diabetes.

## Author Contributions

HK and DX were responsible for study design and data acquisition. SG and ZJ contributed to data analysis. YG and YW participated in data acquisition. DX and HW wrote and all the authors revised and approved the final manuscript.

### Conflict of Interest Statement

The authors declare that the research was conducted in the absence of any commercial or financial relationships that could be construed as a potential conflict of interest.

## Supplementary Material

Supplementary Figure 1 Insulin immunohistochemistry and electron microscope images of pancreatic β cells of male fetal rats induced by prenatal ethanol exposure (PEE). **(A–D)** Representative images of pancreatic insulin immunohistochemical staining (×40 and ×400), and **(E,F)** electron microscope images of pancreatic β cells. *n* = 5 for insulin immunohistochemistry.

Supplementary Figure 2 Insulin immunohistochemistry of prenatal ethanol exposure (PEE) male offspring rats at postnatal week (PW) 6, PW12 and PW24.**(A–D)** Representative images of insulin immunohistochemical staining at PW6 (×40 and ×400); **(E–H)** representative images of insulin immunohistochemical staining at PW12 (×40 and ×400); **(I–L)** representative images of insulin immunohistochemical staining at PW24 (×40 and ×400).

Supplementary Figure 3 Glucocorticoid receptor (GR), insulin-like growth factor (IGF1) and insulin gene enhancer protein isl1 (ISL1) immunohistochemistry of prenatal ethanol exposure (PEE) male offspring rats at postnatal week (PW) 12 and PW24. **(A,B)** Representative images of GR at PW12, **(C,D)** representative images of IGF1 at PW12, **(E,F)** representative images of ISL1 at PW12, **(G,H)** representative images of GR at PW24, **(I,J)** representative images of IGF1 at PW24, and **(K,L)** representative images of ISL1 at PW24.

Supplementary Figure 4 Glucocorticoid receptor (GR), insulin-like growth factor (IGF1), insulin gene enhancer protein isl1 (ISL1) and INSULIN immunohistochemistry of prenatal ethanol exposure (PEE) male offspring rats at postnatal week (PW) 12 with chronic stress (CS). **(A,B)** Representative images of GR, **(C,D)** representative images of IGF1, **(E,F)** representative images of ISL1, and **(G,H)** representative images of INSULIN.
