# Supplementary figures and images for: Age-Characteristic Changes of Glucose Metabolism, Pancreatic Morphology and Function in Male Offspring Rats Induced by Prenatal Ethanol Exposure
Source: Front Endocrinol (Lausanne). 2019 Feb 4;10:34. doi: 10.3389/fendo.2019.00034 (PMC6369175; doi:10.3389/fendo.2019.00034)

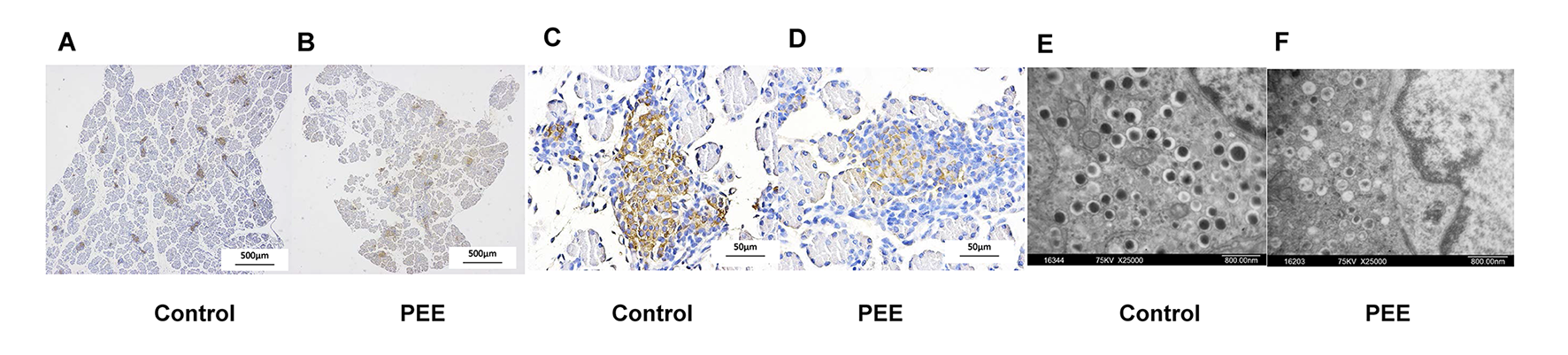

Supplement: Supplementary Figure 1 — Insulin immunohistochemistry and electron microscope images of pancreatic β cells of male fetal rats induced by prenatal ethanol exposure (PEE). (A–D) Representative images of pancreatic insulin immunohistochemical staining (×40 and ×400), and (E,F) electron microscope images of pancreatic β cells. n = 5 for insulin immunohistochemistry. [file Image_1.TIF]

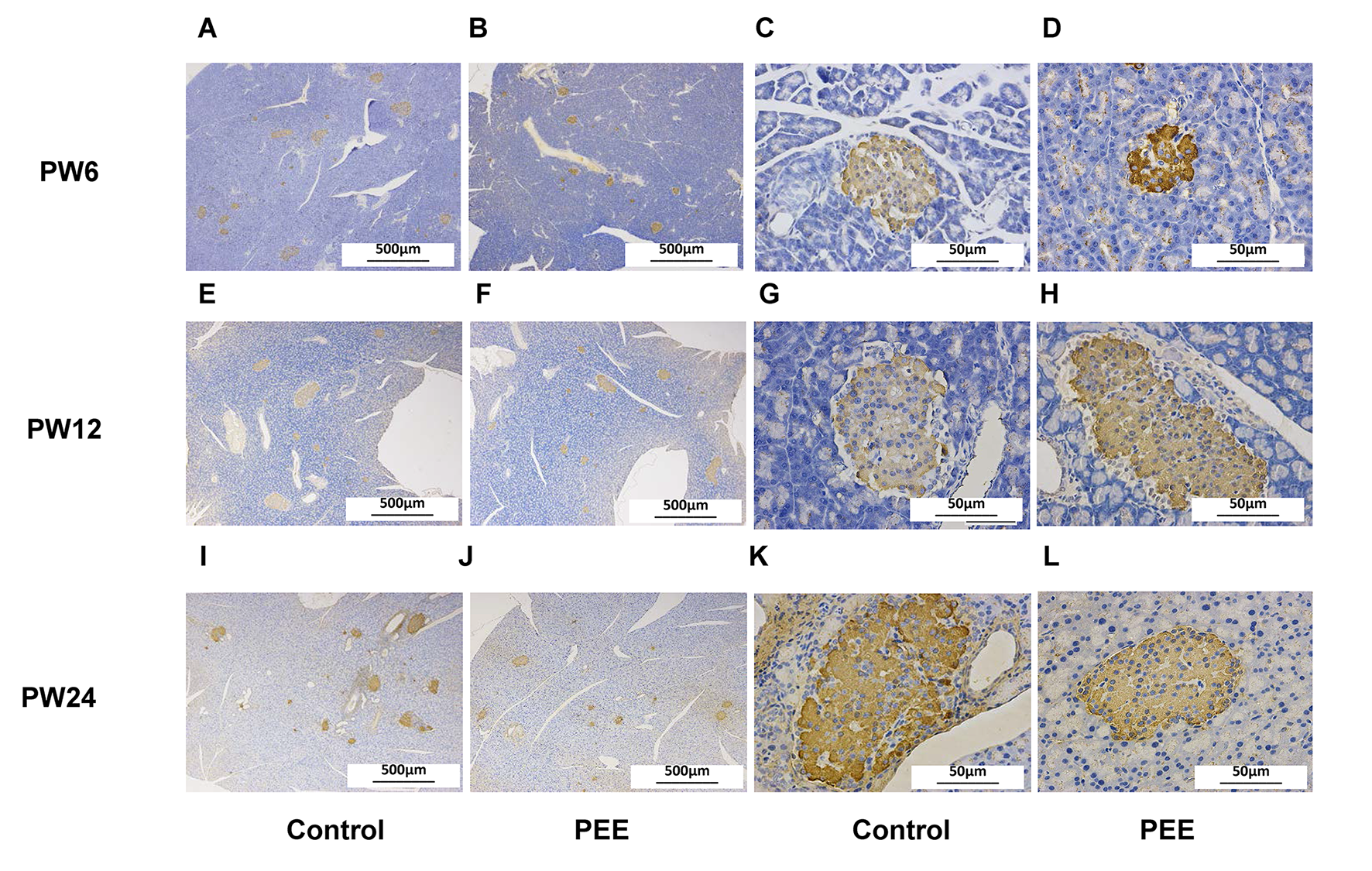

Supplement: Supplementary Figure 2 — Insulin immunohistochemistry of prenatal ethanol exposure (PEE) male offspring rats at postnatal week (PW) 6, PW12 and PW24.(A–D) Representative images of insulin immunohistochemical staining at PW6 (×40 and ×400); (E–H) representative images of insulin immunohistochemical staining at PW12 (×40 and ×400); (I–L) representative images of insulin immunohistochemical staining at PW24 (×40 and ×400). [file Image_2.TIF]

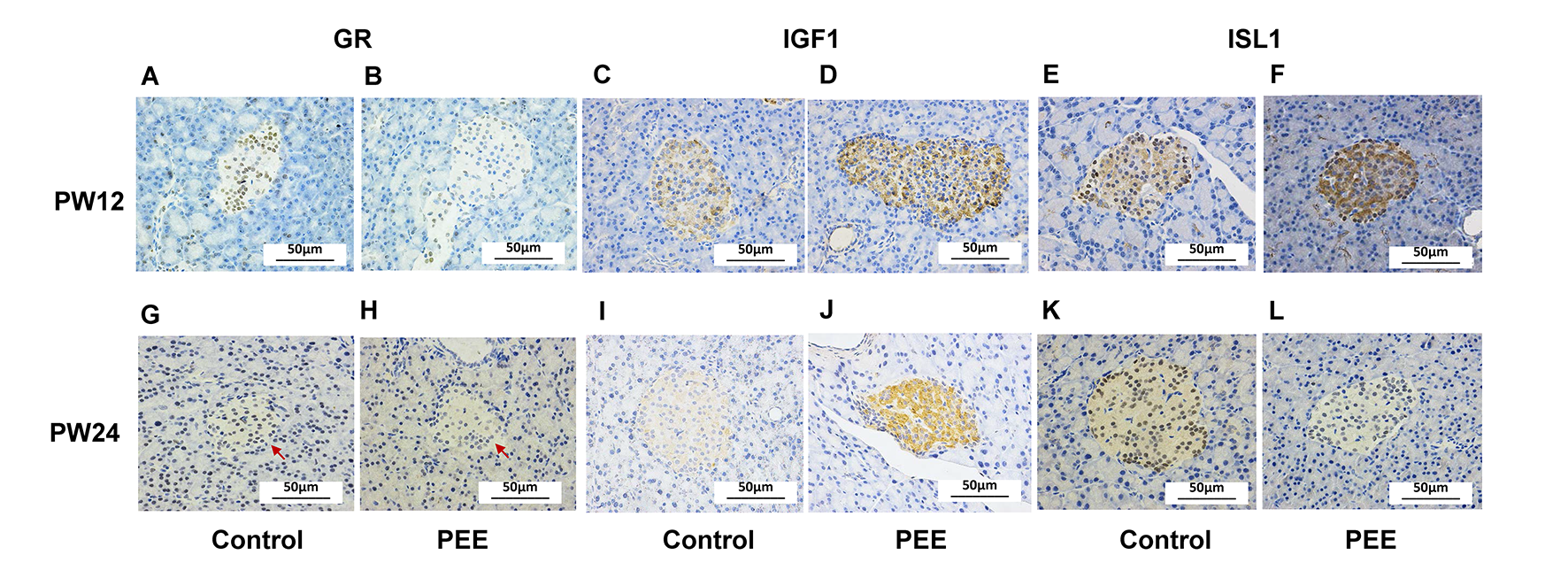

Supplement: Supplementary Figure 3 — Glucocorticoid receptor (GR), insulin-like growth factor (IGF1) and insulin gene enhancer protein isl1 (ISL1) immunohistochemistry of prenatal ethanol exposure (PEE) male offspring rats at postnatal week (PW) 12 and PW24. (A,B) Representative images of GR at PW12, (C,D) representative images of IGF1 at PW12, (E,F) representative images of ISL1 at PW12, (G,H) representative images of GR at PW24, (I,J) representative images of IGF1 at PW24, and (K,L) representative images of ISL1 at PW24. [file Image_3.TIF]

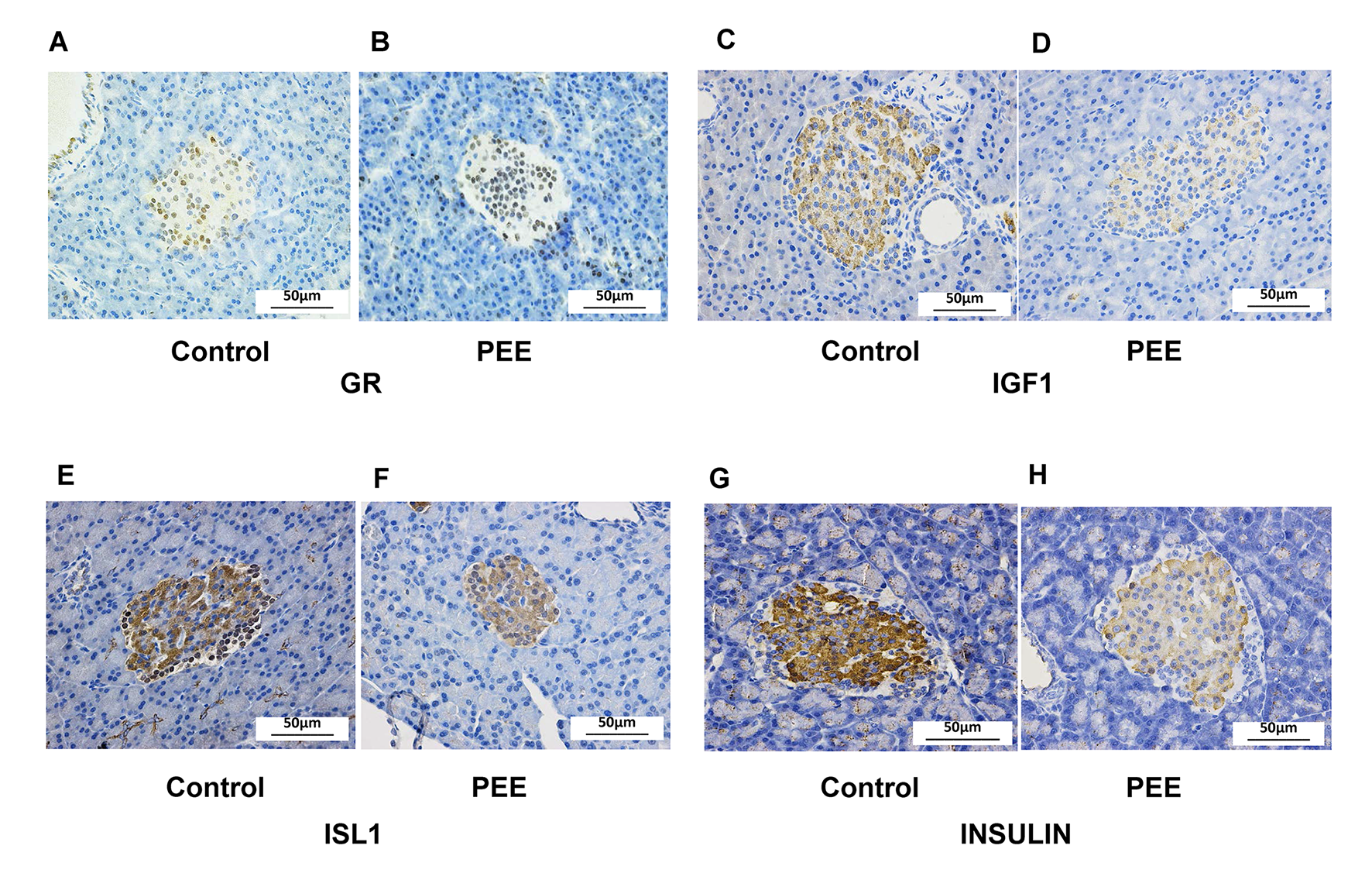

Supplement: Supplementary Figure 4 — Glucocorticoid receptor (GR), insulin-like growth factor (IGF1), insulin gene enhancer protein isl1 (ISL1) and INSULIN immunohistochemistry of prenatal ethanol exposure (PEE) male offspring rats at postnatal week (PW) 12 with chronic stress (CS). (A,B) Representative images of GR, (C,D) representative images of IGF1, (E,F) representative images of ISL1, and (G,H) representative images of INSULIN. [file Image_4.TIF]
